# 3D-Printed PCL/PLA Composite Stents: Towards a New Solution to Cardiovascular Problems

**DOI:** 10.3390/ma11091679

**Published:** 2018-09-11

**Authors:** Antonio J. Guerra, Paula Cano, Marc Rabionet, Teresa Puig, Joaquim Ciurana

**Affiliations:** 1 Department of Mechanical Engineering and Civil Construction, Universitat de Girona, C/Maria Aurèlia Capmany 61, 17003 Girona, Spain; antonio.guerra@udg.edu (A.J.G.); m.rabionet@udg.edu (M.R.); 2 Department of Medical Sciences, Faculty of Medicine, University of Girona, Emili Grahit 77, 17003 Girona, Spain; paaula.cano@gmail.com (P.C.); teresa.puig@udg.edu (T.P.)

**Keywords:** 3D-printing, stent, bioabsorbable, bioresorbable, polymer, composite

## Abstract

Biodegradable stents (BRS) offer enormous potential but first they must meet five specific requirements: (i) their manufacturing process must be precise; (ii) degradation should have minimal toxicity; (iii) the rate of degradation should match the recovery rate of vascular tissue; (iv) ideally, they should induce rapid endothelialization to restore the functions of vascular tissue, but at the same time reduce the risk of restenosis; and (v) their mechanical behavior should comply with medical requirements, namely, the flexibility required to facilitate placement but also sufficient radial rigidity to support the vessel. Although the first three requirements have been comprehensively studied, the last two have been overlooked. One possible way of addressing these issues would be to fabricate composite stents using materials that have different mechanical, biological, or medical properties, for instance, Polylactide Acid (PLA) or Polycaprolactone (PCL). However, fashioning such stents using the traditional stent manufacturing process known as laser cutting would be impossible. Our work, therefore, aims to produce PCL/PLA composite stents using a novel 3D tubular printer based on Fused Deposition Modelling (FDM). The cell geometry (shape and area) and the materials (PCL and PLA) of the stents were analyzed and correlated with 3T3 cell proliferation, degradation rates, dynamic mechanical and radial expansion tests to determine the best parameters for a stent that will satisfy the five strict BRS requirements. Results proved that the 3D-printing process was highly suitable for producing composite stents (approximately 85–95% accuracy). Both PCL and PLA demonstrated their biocompatibility with PCL stents presenting an average cell proliferation of 12.46% and PLA 8.28% after only 3 days. Furthermore, the PCL/PLA composite stents demonstrated their potential in degradation, dynamic mechanical and expansion tests. Moreover, and regardless of the order of the layers, the composite stents showed (virtually) medium levels of degradation rates and mechanical modulus. Radially, they exhibited the virtues of PCL in the expansion step (elasticity) and those of PLA in the recoil step (rigidity). Results have clearly demonstrated that composite PCL/PLA stents are a highly promising solution to fulfilling the rigorous BRS requirements.

## 1. Introduction

Biodegradable stents (BRS) were introduced to overcome the limitations of permanent stents and to offer significant advantages such as, among others, complete bioresorption and/or mechanical flexibility. [1]. BRS have the potential to improve long-term patency rates by providing support just long enough for the artery to heal. Ideally, BRS should meet some exacting requirements.

A large number of authors have contributed to BRS research in recent years. For instance, in terms of manufacturing processes laser cutting and, more recently, additive manufacturing technologies have been at the center of many studies. Guerra et al. [2] studied the effects laser cutting has on the degradation rate of Polycaprolactone (PCL) stent subunits under dynamic and static conditions. Although their results showed that laser cutting has a negligible effect on degradation, the degradation conditions showed that PCL degrades faster in body conditions (dynamics) than the data found in traditional literature had reported (statics). Meanwhile, Grabow et al. [3] studied the effects CO_2_ laser cutting and sterilization have on Poly-L-Lactide (PLLA). Their results revealed the enormous influence sterilization has on the mechanical properties of PLLA (i.e., 40% crystalline modification). Using additive manufacturing, Park et al. [4] produced drug-coated BRS with Fused Deposition Modelling (FDM) and had very promising results in animals (20.7% restenosis). Guerra et al. [5,6] designed and implemented a novel 3D tubular printer that allows the rapid manufacture of BRS based on polymers. Their results suggest that this technology could be the future of BRS manufacturing as they managed to achieve up to 85% precision and manufacturing times under 5 min.

In terms of the abovementioned second (degradation should have minimal toxicity), third (the rate of degradation should match the recovery rate of vascular tissue), and fifth (mechanical behavior should meet medical stipulations) requirements, some authors have focused their studies on potentially suitable materials for producing BRS [7,8]. Hideo Tamai et al. [9] evaluated the feasibility, safety, and efficacy of PLLA stents in humans for coronary artery stenosis with promising results. Shen et al. [10] studied the degradation of L-Lactide (LA) and 5-methyl-5-benzyloxycarbonate-1,3-dioxan-2-one (MBC) coated stents and cast films. Their results showed similar degradation behavior of the coating materials in in vivo conditions and negligible differences in extensive endothelialization or the expression of inflammation-associated proteins after 4 weeks post-stent implantation.

However, there are fewer studies that consider the fourth requirement (BRS should induce rapid endothelialization to restore the functions of vascular tissue but, at the same time, reduce the risk of restenosis). Wang et al. [11] studied the cell adhesion of Human Umbilical Vein Endothelial Cells (HUVEC) on a stent coated with poly-l-lysine and fibronectin. Their work revealed that the metallic-coated stent significantly increased cell adhesion. Meanwhile, Xu et al. [12] developed strategies for improving stent endothelialization by employing a new polymer poly-1,8-octanediol-co-citric acid (POC), anti-CD34 antibody and a vascular endothelial growth factor. Their method significantly improved the proliferation of endothelial progenitor cells (EPC) when compared with PLLA stents. In 2015, Lutter et al. [13] studied the influence the microstructure of a stent’s surface has on endothelialization and thrombogenicity using HUVEC and their results demonstrated that, compared to a smooth surface stent, flat cubic elevation improved endothelial cell adhesion. Additionally, Jiang et al. [14] analyzed four polymer coatings for controlling the degradation and HUVEC cell adhesion of Mg stents. Employing PLLA, poly-lactic-*co*-glycolic-acid (PLGA) (90:10), PLGA (50:50), and PCL, they evaluated surface and biological properties. Their results showed that PLGA (50:50) is a promising coating material for Mg stents. Finally, He et al. [15] studied how to design nanofiber mesh which would allow the attachment and phenotypic maintenance of human coronary artery endothelial cells. Similar work was carried out by Rubert M. et al. [16], who analyzed coaxial electrospun with PCL materials and added poly(ethylene oxide) (PEO) fibers containing basic fibroblast growth factor. The work also focused on fibroblast proliferation by combining cell culture and proliferation with additive manufacturing technologies.

However, many challenges still exist, such as evaluating and understanding the mechanical and biological properties of polymeric BRS. Previous work carried out by our groups [17] has proved that composite stents satisfy some of the requisites. However, composite stents cannot be created easily with conventional laser cutting manufacturing processes [18]. Alternative manufacturing technologies, such as 3D-printing, need to be used. This work aims to develop PCL/PLA (Polylactide Acid) composite stents by employing 3D-printing based on FDM. Both the parameters of the stent, namely, its cell geometry (shape and area) and the materials (PCL and PLA), were analyzed. Stents were subjected to 3T3 cell proliferation, degradation, dynamic mechanical, and radial expansion tests to determine which parameters would best comply with the stringent BRS requirements. This work presents a proof of concept for the viability of using composites stents in the treatment of cardiovascular disease.

## 2. Materials and Methods

### 2.1. Stent Fabrication Method

**Figure 1:**
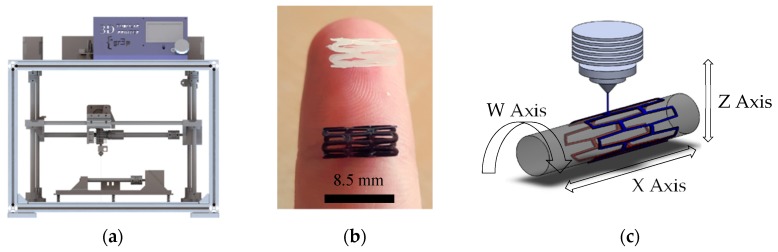
(**a**) 3D Tubular printer (**b**) 3D-printed stents [PCL in white, PLA in black] (**c**) Machine methodology. PCL: Polycaprolactone; PLA: Polylactide Acid.

Stents were manufactured by employing a 3D-printing process in a 3D tubular printer [5]. This 3D additive manufacturing machine is based on the FDM method. The filament is melted into the extruder nozzle, which then deposits the melted material onto a controlled rotatory platform. The machine provides 0.9375 µm precision on the X axis, 0.028125° on the W axis, 0.3125 µm on the Z axis, 0.028125° in the extruder that has a 0.4 mm diameter nozzle (Figure 1).

**Figure 2:**
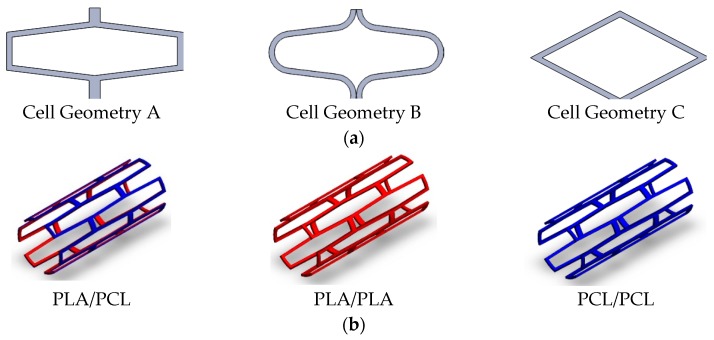
Stent configurations: (**a**) Stent cell geometries employed; (**b**) Stent material/layers used.

Stents are defined by their cell geometry shape (C_G_:A:C), inner diameter (I_Ø_: 4 mm), stent thickness (S_T_: 0.2 mm), number of circumferential cells (N_C_: 4:8), cell area (C_A_), strut width (S_W_), total length (L: 9.6 mm), and material (M_a_:PCL:PLA) (Figure 2). Based on previous work [6], the PCL stents were printed at a temperature of 220 °C for nozzle Tª, 25 °C for bed Tª, and a printing speed of 300 mm/min, while for the PLA stents, the temperature was 220 °C for nozzle Tª, 25 °C for bed Tª, and a printing speed of 200 mm/min printing speed. Different printing flow rate percentages (F_R_) were also employed for each material to obtain different C_A_.

To study the effects the geometrical and material aspects of the stents have on their final properties, 27 geometries using different combinations of C_G_, N_C_, F_R_, and M_a_ with a Full Factorial Design (FFD) were printed (Table 1). Stents were sterilized overnight in a 70% ethanol/water solution, then washed twice with Phosphate Buffered Solution (PBS, Gibco, Walthman, MA, USA) and finally exposed to UV light for 30 min. This sterilization method was followed to avoid any changes in the final properties of the stents [19].

### 2.2. Materials

The materials used were Polycaprolactone CAPA 6500^®^ (PCL, Perstorp, Sweden) and Polylactide Acid (PLA, RepRap BCN) (Table 2). PCL is a biodegradable polyester with a low melting point (60 °C) and has a glass transition of about −60 °C. Meanwhile, PLA is a biodegradable thermoplastic aliphatic polyester derived from renewable resources, such as corn starch or sugarcane, and has a melting point of about 173–178 °C with a glass transition of 60–65 °C. Degradation of both PCL and PLA is produced by the hydrolysis of their ester linkages in physiological conditions. White PCL and black PLA were selected so as to easily identify the materials once the printing process had been completed.

### 2.3. Cell Line

Murine 3T3 fibroblasts cells were obtained from the American Type Culture Collection (ATCC, Rockville, MD, USA). The 3T3 cells were cultured in Dulbecco’s Modified Eagle’s Medium (DMEM; Gibco, Walthman, MA, USA) supplemented with 10% fetal bovine serum, 1% L-glutamine, 1% sodium pyruvate, 50 U/mL penicillin and 50 μg/mL (HyClone, Logan, UT, USA). Cells were kept at 37 °C and 5% CO_2_ atmosphere. To obtain more reliable results, a total of 12 replicas for both materials [6 for PCL (3 × 2) and 6 for PLA (3 × 2)] were tested. Two-dimensional (2D) cell culture was also performed to normalize the cell proliferation rates on the stents. Because of their rapid and stable growth kinetic, fibroblast cells were selected to carry out the first proof of concept for the PCL/PLA composite stents. However, further studies with other kinds of cells, such as endothelial human cell, are required and should be performed.

### 2.4. Culture Methodology

The stents were placed in 24-well non-adherent microplates (Sartstedt, Nümbrecht, Germany), soaked with DMEM and incubated at 37 °C prior to cell seeding. Finally, due to cell growth kinetics and the culture period, the stents were seeded with a 20,000-cell-per-stent concentration and kept at 37 °C in a 5% CO_2_ atmosphere for 3 days. Because our aims were to determine whether PCL and PLA are biologically valid and to define the stent geometry that produces the best cell proliferation, cell culture was performed in static conditions. However, further work using dynamic conditions to simulate a real environment would be beneficial because under dynamic conditions stents would be subjected to pressure and flow conditions that can modify the kinetics of cell proliferation.

### 2.5. Characterization

#### 2.5.1. Morphological Features

A Nikon SMZ-745T optical microscope (Nikon, Minato, Tokyo, Japan) attached to a ProgRes CT3 digital camera (Jenoptik, Jena, Germany) was used to determine the dimensional features (precision, layer adhesion, etc.) of the samples. Image J^®^ (National Institutes of Health, Bethesda, MD, USA) was used to process the images and collect the data.

#### 2.5.2. Cell Proliferation Assay

MTT tests were performed to check the effect the process parameters (C_G_, N_C_, F_R_, and M_a_) had on cell proliferation. The subsequent results allowed us to then select the best parameters with which to manufacture the final PCL/PLA composite stents with the best biological response. An MTT (3-4.5-dimethyl-2-thiazolyl)-2.5-diphenyltetrazolium bromide) assay was selected to test the cell proliferation on the stents. MTT is a yellow tetrazolium salt which can be reduced by the mitochondria of viable cells. This metabolic transformation results in water-insoluble purple crystals of formazan being produced, which can then be solubilized with dimethyl sulfoxide (DMSO) into a colored solution. The absorbance of DMSO solution is related to the number of cells. After incubation, the culture medium was removed, and the stents were put into new wells where they were incubated with 3 mL of DMEM medium and 300 µL of MTT (Sigma-Aldrich, Saint Louis, MO, USA) for 2.5 h. After this time, the MTT supplemented culture medium was removed, DMSO was added and the stents were shacked to ensure the complete dissolution of the resulting formazan crystals. Four 100 µL aliquots from each sample were pipetted into a 96-well plate and absorbance was measured at 570 nm using a microplate reader (Bio-Rad, Hercules, CA, USA). Adherent 2D controls were likewise processed with the same number of seeded cells.

#### 2.5.3. Cell Adhesion Assay

To check the cell adhesion on the stents, Confocal Laser Microscopy was performed with a Nikon A1R Microscope. The cultivated samples were dyed as follows: (i) wash samples with phosphate buffered solution (PBS, Gibco, Walthman, MA, USA); then (ii) fix samples with 4% paraformaldehyde at room temperature and wash samples; next (iii) permeabilize cells with 0.2% Triton X-100; and (iv) add blocking buffer at room temperature; then (v) dye cytoskeleton actin with rhodamine-phalloidin (Cytoskeleton Inc., Denver, CO, USA)(1:200) in blocking buffer; and finally (vi) dye the cells nuclei with (4’,6-diamidino-2-phenylindole) (DAPI) (1/1000) in blocking buffer. The adhesion assay was performed simply to obtain qualitative data about cell adhesion.

#### 2.5.4. Mechanical Properties

The PCL, PLA, and PCL/PLA samples were subjected to Dynamic Mechanical Analysis using a METTLER TOLEDO SDTA861e Dynamic Mechanical Analyzer (DMA, METTLER TOLEDO, Columbus, OH, USA). The dynamic storage modulus (*E’*) was analyzed. The loss tangent is the ratio of loss modulus and storage modulus and this indicates the material’s viscosity and elastic properties, respectively. The loss tangent curve peak appears and can be defined as the material’s glass transition temperature ($T_{g}$).

#### 2.5.5. Degradation Rate

The PCL, PLA, and PCL/PLA samples were submerged in phosphate buffered solution (PBS, 7.4 ± 1 pH) at 37 °C for 6 weeks. Samples were recovered after 2, 4, and 6 weeks under the same conditions (25 °C). Using a METTLER TOLEDO Sartorius 2MP Scale (METTLER TOLEDO, Columbus, OH, USA), weight loss was evaluated by measuring the original weight of the sample after the printing process (*W*_0_), and its residual weight after degradation and once it had been completely dried (*W_r_*) in an oven at 30 °C for 24 h. Weight loss percentage, *W_L_*%, was estimated using the following equation:

(1) $$\;W_{L}\%=\frac{W_{0}-W_{r}}{W_{0}}\times 10\;$$

The PBS was changed frequently to keep the pH as constant as possible.

#### 2.5.6. Radial Behavior

The PCL, PLA, and PCL/PLA samples were expanded radially from their original diameter (O_Ø_: 4 mm) until a maximum of 175% of their original diameter (M_Ø_: 7 mm) was reached. Then, the radial force was removed, and the diameter was measured to determine the recoil ratio (R_Ø_).

#### 2.5.7. Statistics

Results were subjected to regression analysis. The analysis of variance (ANOVA) method was applied to test the statistical significance of the process parameters (C_G_, N_C_, F_R_, and M_a_). The analysis was carried out at a 95% confidence level (α = 0.05). All observations were confirmed by at least three independent experiments. All data are expressed as mean ± standard error (SE).

## 3. Results

**Figure 3:**
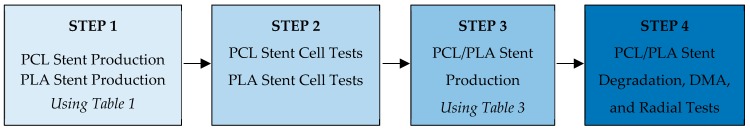
Methodology followed to carry out the experiments. DMA: Dynamic Mechanical Analyzer.

In this manuscript, the following steps were executed to perform the experiments (Figure 3).

### 3.1. Cell Proliferation Results

**Figure 4:**
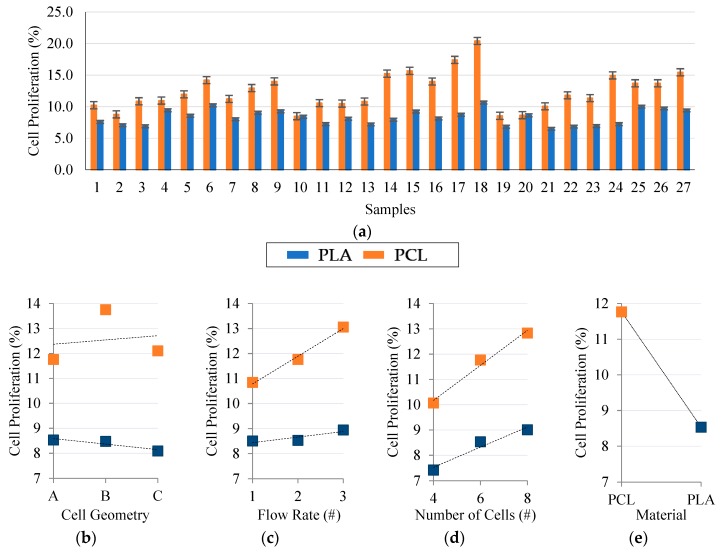
3T3 Proliferation Results: (**a**) Average cell proliferation on each sample; (**b**) Main effect of cell geometry (6 radial cells, 2nd flow rate); (**c**) Main effect of flow rate (6 radial cells, A Geometry); (**d**) Main effect of number of cells (A Geometry, 2nd flow rate); (**e**) Main effect of plot of materials (6 radial cells, A Geometry, 2nd flow rate).

Cell proliferation is mainly related to the molecular weight, stent geometry and the intrinsic porosity of the material [20]. This section presents the effects these parameters had on the final cell proliferation. Murine 3T3 fibroblast culture results (Figure 4a) showed the different biological responses of PCL and PLA. Regardless of the geometrical aspects of the stent, the PCL stents showed a 33.5% greater cell proliferation compared to the PLA stents.

When analyzing the results in terms of input parameters (C_G_, N_C_, F_R_, and M_a_), regression analysis showed the considerable statistical influence that flow rate, number of stent radial cells, and material (*p* < 0.05) have on cell proliferation. Stent cell geometry did not show any significant statistical influence on cell proliferation (Figure 4b); however, we hypothesize that geometries with a higher number of pointed corners might well improve cell adhesion and subsequent proliferation. Some previous studies made by others have demonstrated that stent geometry affected the neointima cell proliferation and exerted a substantial influence on thrombosis and restenosis rates [21].

The increase in flow rate and number of cells produced the increase in cell proliferation (Figure 4c,d). This increase is motivated by the reduction in the stent’s cell area. The cell area (pore size) of a stent has been recognized as an important parameter that affects the proliferation properties and functions of the scaffolds (stents) because it is directly related to cell migration, vascularization, and mass transport [22]. Moreover, a high flow rate value implies a greater amount of expelled material. Therefore, cells have more material to adhere to and spread to, thus increasing their proliferation rate. Likewise, decreasing the number of stent cells resulted in stents with more material, thus allowing the proliferation of more cells.

Finally, in terms of the decisive effects materials have, lower molecular weights are known to produce poorer cell proliferation [23]. Here, PCL had a M_W_ of 50,000 g/mol, which is 40% more than PLA had at 30,000 g/mol. This percentage difference closely resembles that obtained in the proliferation results (PCL was 33.5% greater than PLA).

**Figure 5:**
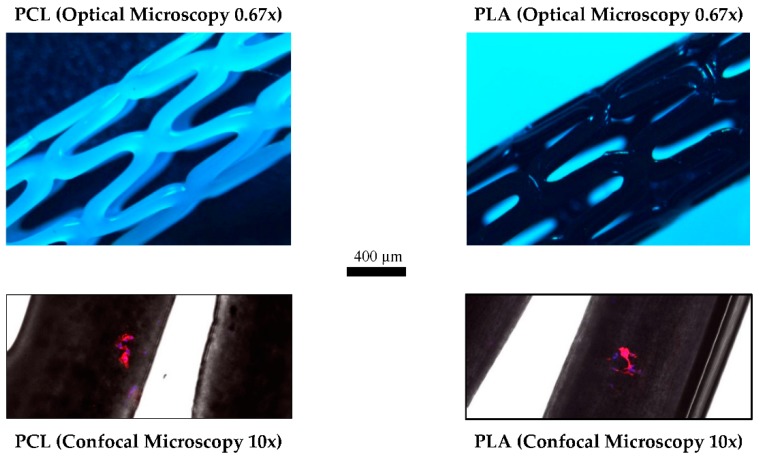
Confocal Laser Microscopy Images: (**Left**) PCL stent; (**Right**) PLA stent. Samples cultured with 3T3 fibroblast cells. Nucleus was stained with DAPI (blue) and actin cytoskeleton was stained with rhodamine-phalloidin (red).

Confocal Laser Microscopy was performed on the PCL and PLA samples to qualitatively determine how 3T3 cells stick to the stents (Figure 5). The objective of this test was to study where the majority of cells adhere to and thus to be able to design stents that produce better proliferation. Figure 5 suggests that cells possess the ability to adhere to any part of the stent, even so, they usually adhered close to the pores and the corners of the stent. Further studies using different stent geometries could be useful to determine how to improve cell proliferation.

The results prove the materials’ biological compatibility and encourage us to believe that PCL/PLA composite stents would comply with the fourth requirement, i.e., rapid endothelization without risk of restenosis. PCL’s better cell proliferation may be useful to increase the proliferation of endothelial vessel cells in the external wall of the stents, while an internal PLA wall may help to reduce the proliferation of cells that produce restenosis. However, further studies with other kinds of cells or substances need to be performed to confirm this. The results here show low cell proliferation because of the small amount of material that the stents have. Additional studies that use longer culture times may be beneficial to obtain better proliferation results.

### 3.2. Composite PCL/PLA Stent Manufacturing Results

As the properties of the stents are interrelated and sometimes contradictory, they require a careful compromise. For example, stents should be flexible enough to facilitate their placement but rigid enough to support the vessel. Sometimes higher tensile strength materials, desirable for bolstering radial strength, have higher yield strengths that promote undesired acute recoil. Biological aspects are also contradictory. For example, scaffolds (stents) with smaller pores (cell areas) will give better cell proliferation [24,25]; however, these characteristics will induce poorer radial behavior and vessel support. Therefore, and despite the best cell proliferation being obtained with the 18th DOE configuration (Table 1), other manufacturing parameters were used (Table 3) to produce PCL/PLA composite stents that reach a compromise between biological and mechanical properties. The inner diameter (I_Ø_: 4 mm), stent thickness (S_T_: 0.3 mm [0.15 mm/layer]), total length (L: 19 mm), printer nozzle Tª (220 °C), printer bed Tª (25 °C), printer infill (100%), and printer speed (300 mm/s for PCL, 200 mm/s for PLA) were kept constant. All the layer sequences were tested to identify if they influenced the properties or not.

**Figure 6:**
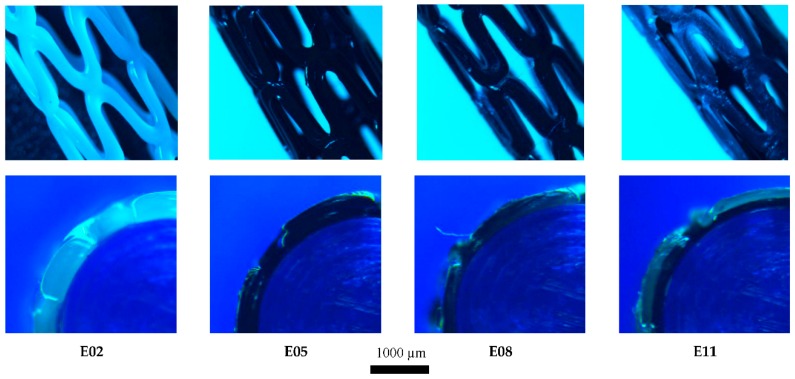
Optical Nikon Microscope images of 3D-printed stents. Superior images show the general 3D view, while inferior images depict the ¼ section radial view. Samples numbered according to Table 3. PCL/PLA composite stent fabrication parameters.

The manufacturing process successfully produced PCL/PLA stents (Figure 6), requiring only 3 min per sample and achieving approximately 85–90% accuracy (depending on the geometry of the stent in question).

The intrinsic properties of 3D-printing based on FDM, make producing an object with pointed corners (geometries A and C) impossible. However, the rounded corners obtained could be useful when stents are being implanted and/or might reduce any potential damage to the endothelial in the vessel wall.

### 3.3. Degradation Rate Results

The mechanisms causing PCL and PLA degradation are well known: degradation occurs when water penetrates the bulk of the material and causes hydrolysis throughout the entire polymer matrix. From the literature it can be concluded that PCL and PLA undergo a two-stage degradation process: first, the non-enzymatic hydrolytic cleavage of ester groups and, second, when the polymer is more highly crystalline and of low molecular weight it experiences intracellular degradation and may be completely resorbed and degraded via an intracellular mechanism [26].

**Figure 7:**
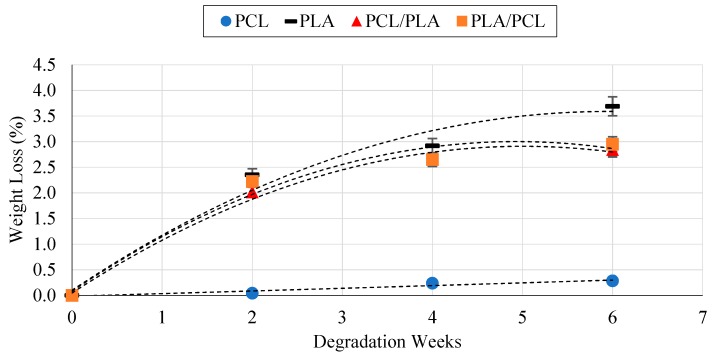
Degradation rate results for PCL, PLA, PCL/PLA, and PLA/PCL stents.

Degradation tests considering all the possible layer configurations were performed to establish if the sequence of layers affected the degradation rate or not. Simple PCL/PLA sheets were printed and tested (leaving aside the effect the stent’s cell geometry (C_G_, N_C_, and F_R_) would have). Results have evidenced the variations in degradation rates between PCL and PLA (Figure 7). This difference is produced by the difference in their molecular weights [19]. As is well known, materials with lower molecular weights degrade faster. However, both the PCL/PLA and the PLA/PCL composite stents showed almost medium weight losses, making the composite stent a good option in terms of degradation. Results also proved that the order in which the layers are placed has no effect on the device’s degradation rate. However, in real conditions the outermost surface would be placed against the blood vessel wall which could, in turn, produce faster degradation. This is something which should be investigated further.

### 3.4. Dynamics Mechanical Analysis

**Figure 8:**
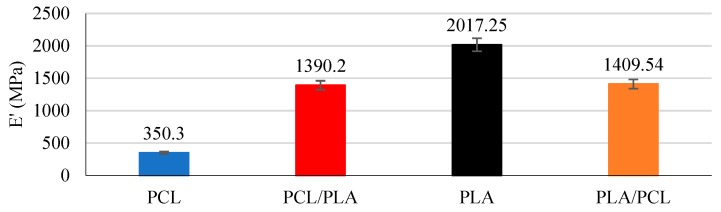
DMA results for PCL, PLA, PCL/PLA and PLA/PCL stents.

Again, all the configurations of the layers were tested to determine whether they affected the mechanical behavior of the stents in any way or not. For the DMA tests, simple PCL/PLA sheets were printed and tested (leaving aside the effect the stent’s cell geometry has (CG, NC, and F_R_)). Results (Figure 8) showed the different mechanical behaviors of PCL and PLA and make it clear that alone they cannot be used. Nevertheless, composite PCL/PLA stents have shown a middle *E’* that would be more appropriate for stent manufacture [12]. As with the degradation rates, the order of the layers did not show any influence on the dynamic modulus.

### 3.5. Radial Behavior Results

The radial behavior of stents is one of the most important properties because this is what provides the vessel with the appropriate support it needs. The effects the process parameters (C_G_, N_C_, F_R_, and M_a_) and the composition of the layers have on the radial behavior were analyzed.

**Figure 9:**
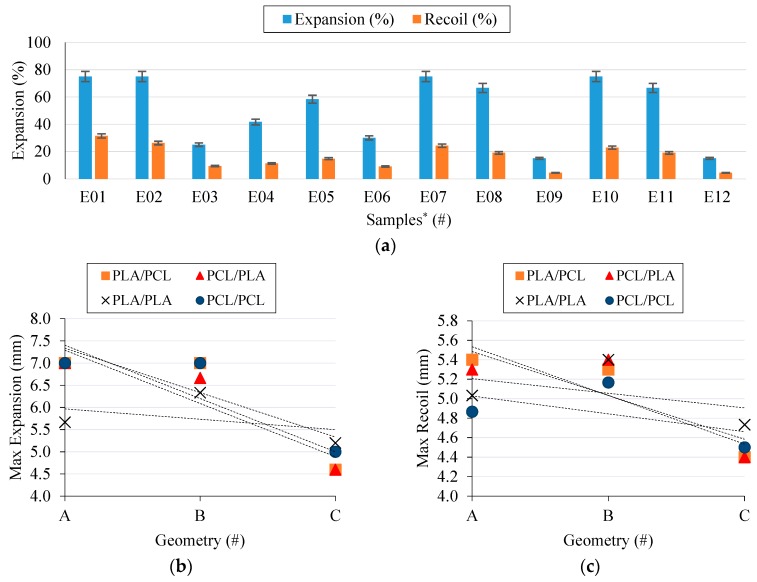
Radial expansion behavior results: (**a**) Expansion and recoil of each sample, (**b**) Main effect plot of geometry and layer order on radial expansion, (**c**) Main effect plot of geometry and layer order on radial recoil. * Samples numbered according to Table 3. PCL/PLA composite stent fabrication parameters.

Unlike in the cell proliferation results, stent cell geometry proved to exert a considerable influence on the radial expansion behavior (Figure 9a,b). The A geometries, with their higher hinges, provided the best results. However, the higher the hinge, the greater the recoil (Figure 8c), which is something that could negatively affect the placement procedure of the stent.

PCL stents (Figure 9a E01 → E03) showed the best radial expansion results; nevertheless, the elasticity of PCL produced higher recoil ratios, which is an undesirable effect. PLA stents (Figure 9a E04 → E06) showed an excellent recoil ratio but an unacceptable radial expansion. Meanwhile, composites stents with either an inner layer of PCL (Figure 9a E07 → E09) or of PLA (Figure 9a E10 → E12) showed very interesting results. Layer order did not affect the radial expansion behavior and exhibited the same results for stents with the same cell geometry. Composite stents exhibited the characteristics of PCL stents in the expansion process as well as the benefits of PLA stents in the recoil. Thanks to the elasticity of PCL, the PLA layer remained unbroken in the expansion process. Furthermore, when the force was removed, the rigidity of PLA hindered the recoil of PCL.

Results confirm that composite stents can improve the mechanical properties of materials. Their good radial behavior, regardless of the order the layers are placed in, make it possible to produce composites stents with an inner layer of PLA and an outer layer of PCL that comply with the strict BRS requirements.

## 4. Conclusions

To our knowledge, this is the first work to develop and present composite PCL/PLA stents using a 3D-printing process based on FDM. The effects the cell geometry (shape and area) and materials (PCL and PLA) exert were also analyzed. Samples were subjected to 3T3 cell proliferation, degradation, dynamic mechanical and radial expansion tests to determine the parameters that best meet the rigorous requirements for BRS.

The results have demonstrated the considerable influence the cell area and material of a stent have on 3T3 proliferation. That said, the cell shape of the stent did not show any significant influence at all. Our initial hypothesis was confirmed, i.e., the smaller the cell area of a stent, the better the cell proliferation rate. Meanwhile, as a result of their different molecular weights, PCL demonstrated better cell proliferation than PLA.

The degradation rate results revealed the limitations of PLA for BRS purposes as a consequence of its fast degradation rate, whereas PCL showed a better degradation rate. The composite PCL/PLA stents showed an almost medium degradation for all the layer configurations which was mainly due to PLA degradation. The faster PLA degradation rate would eventually leave a BRS made only of PCL. The differences in their degradation rates are produced by their different molecular weights, so employing PCL and PLA with similar molecular weights is a must to obtain a homogenous degradation of all the layers.

PCL showed a very low *E’* modulus in the dynamic mechanical results which, in turn, hinders its sole applicability for stent purposes. On the other hand, PLA showed a high *E’* modulus and good properties for supporting the artery vessel once in place but its properties hinder its placement. Conversely, composite stents showed a middle *E’* modulus regardless of the order the layers were made up of.

Finally, the radial behavior results have proved that composite PCL/PLA stents could be used to improve each material’s separate limitations. For instance, PCL stents presented overly high recoil ratios but excellent expansion behavior, whereas PLA stents presented inadequate radial expansion, due to their rigidity, but excellent recoil ratio. Composite stents, either with PCL or PLA as the inner layer, demonstrated the virtues of PCL stents (i.e., their radial expansion) and PLA stents (i.e., their recoil ratio) that could be combined to provide a good solution for BRS. Furthermore, their good radial behavior (regardless of the order of the layers) make composite stents a promising concept for cardiovascular problems.

Based on the results presented here, we believe that polymer composite stents manufactured with 3D-printing processes could be a highly effective solution to the current problems that stents made of polymers have. However, FDA rules currently limit the use of 3D-printed stents in real clinical applications and, although PCL and PLA are FDA-approved materials, there are still open challenges to be met before approval for 3D-printed implantable medical devices can be obtained. This manuscript has presented a potential approach for future applications for stents.

## Figures and Tables

**Table 1:** Design of Experiments (DOE) of the 54 stents manufactured (27 PCL and 27 PLA).

#	C_G_	N_C_	F_R PCL_ *	F_R PLA_ *	#	C_G_	N_C_	F_R PCL_*	F_R PLA_ *	#	C_G_	N_C_	F_R PCL_ *	F_R PLA_ *
01	A	4	50	85	10	B	4	50	85	19	C	4	50	85
02	A	4	65	100	11	B	4	65	100	20	C	4	65	100
03	A	4	80	115	12	B	4	80	115	21	C	4	80	115
04	A	6	50	85	13	B	6	50	85	22	C	6	50	85
05	A	6	65	100	14	B	6	65	100	23	C	6	65	100
06	A	6	80	115	15	B	6	80	115	24	C	6	80	115
07	A	8	50	85	16	B	8	50	85	25	C	8	50	85
08	A	8	65	100	17	B	8	65	100	26	C	8	65	100
09	A	8	80	115	18	B	8	80	115	27	C	8	80	115

* Levels of F_R_ were chosen to obtain the same S_W_ for PCL and PLA. Three different levels were selected to obtain three different C_A_.

**Table 2:** Material properties.

Material (#)	Molecular Weight (g/mol)	Young’s Modulus (MPa)	Strain at Break (%)	Degradation Time (Months)
PCL	50,000	470	700	>24
PLA	30,000	108	3.5	≈12

**Table 3:** PCL/PLA Composite stent fabrication parameters*^.^

#	C_G_	N_C_	F_R PCL_	F_R PLA_	M_a_ Inner Layer	M_a_ Outer Layer
01	A	6	65	-	PCL	PCL
02	B	6	65	-	PCL	PCL
03	C	6	65	-	PCL	PCL
04	A	6	-	100	PLA	PLA
05	B	6	-	100	PLA	PLA
06	C	6	-	100	PLA	PLA
07	A	6	65	100	PCL	PLA
08	B	6	65	100	PCL	PLA
09	C	6	65	100	PCL	PLA
10	A	6	65	100	PLA	PCL
11	B	6	65	100	PLA	PCL
12	C	6	65	100	PLA	PCL

* Manufacturing parameters selected based on previous results [5,6].
